# Effect of a health education program on reduction of pediculosis in school girls at Amphoe Muang, Khon Kaen Province, Thailand

**DOI:** 10.1371/journal.pone.0198599

**Published:** 2018-06-11

**Authors:** Manachai Yingklang, Chatchawan Sengthong, Ornuma Haonon, Rungtiwa Dangtakot, Porntip Pinlaor, Chulaporn Sota, Somchai Pinlaor

**Affiliations:** 1 Department of Parasitology, Faculty of Medicine, Khon Kaen University, Khon Kaen, Thailand; 2 Biomedical Science Program, Graduate School, Khon Kaen University, Khon Kaen, Thailand; 3 Centre for Research and Development of Medical Diagnostic Laboratories, Faculty of Associated Medical Sciences, Khon Kaen University, Khon Kaen, Thailand; 4 Department of Health Education, Faculty of Public Health, Khon Kaen University, Khon Kaen, Thailand; George Washington University School of Medicine and Health Sciences, UNITED STATES

## Abstract

**Background:**

Pediculosis caused by head lice (*Pediculus humanus capitis*) infestation is still an important health problem in schoolchildren, especially girls, worldwide, including in Thailand. Although pediculicidal agents effectively kill head lice, the re-infestation rate is still high. Thus, prevention is an important strategy for any sustainable control program. We aimed to develop and evaluate the efficacy of a health education program for increasing knowledge, changing attitudes and promoting preventive practices to reduce prevalence of pediculosis among school girls in Amphoe Muang, Khon Kaen, northeastern Thailand.

**Methodology:**

Six schools were selected using multistage simple randomization and were allocated into intervention or control groups. A total of 267 girls was enrolled from these schools. A “knowledge, attitude and practice” (KAP) questionnaire, consent forms and health education materials were constructed and tested by experts and in one pilot school before the main investigation. Baseline prevalence of adult lice and nits was determined. The health education package was given only to the intervention group. The KAP questionnaire was re-evaluated at two months after intervention.

**Results:**

At baseline, the prevalence and intensity of head lice infestation, and the KAP scores did not differ significantly between the two groups. After re-evaluation at two months, the KAP score was significantly greater in the intervention group. A significant decrease of the infestation rate from 59% to 44% was observed in the intervention group, whereas infestation increased in the control group (from 56% to 65%). The incidence of new cases in the intervention group (6.14%) was lower than in the control group (12.62%).

**Conclusion:**

These findings indicated that the newly-established health education package is an effective tool for increasing KAP and reducing head lice infestation in school girls. Efforts to combat pediculosis in schoolchildren elsewhere may consider including this, or a similar, health education package in their programs.

## Introduction

Pediculosis, caused by head lice (*Pediculus humanus capitis*) infestation, is common in schoolchildren worldwide. A recent review has reported that more than 12 million girls, especially in the 3-11-year-old range, are infested with these insects [1]. A high prevalence (up to 59%) was found in developing countries and tropical countries, including Thailand [1]. Lice are spread through direct transmission by head-to-head contact with an infested person at school or at home. Indirect transmission can also occur via the sharing of hairbrushes, clothing, hats, towels and other personal items.

Head lice are obligate blood feeders and hence potentially causing anemia in their hosts [2, 3]. The itching induced by the saliva of head lice can affect sleep, resulting in distraction during studying. Scaling of the scalp is a severe consequence associated with chronic lesions and infection with pathogenic bacteria. Pathogens reportedly transmitted by head lice include *Rickettsia prowazekii*, *Bartonella quintana* and *Borrelia recurrentis* [4–7]. However, unlike body lice, transmission of these agents by head lice appears to be very rare.

Given that the International Pediculosis Association of the USA regards a head lice prevalence of over 5% as being of epidemic level [8], prevalences of up to 59% in Thailand [9], indicate a major problem. The highest prevalence of head lice infestation occurs in children, especially in girls [10, 11]. The Education Administration of Thailand requires boys in primary schools to have short haircuts. For this reason, Thai school boys very rarely have pediculosis [12, 13]. The strategies for elimination of head lice infestation are pediculocidal treatment and alternative method, such as health education. Some pediculicidal agents effectively only kill the adult stage, and the remaining nits will lead to re-infestation, maintaining a high prevalence [14]. For instance, the Health Promotion Division, Department of Health of Thailand, has gathered data on prevalence among children in Bangkok and reported a 60.84% re-infestation rate after treatment with 1% gamma benzene hexachloride in 2008 [14]. Alternative methods to reduce the morbidity and prevalence of pediculosis include strategies such as health education to increase knowledge, change attitudes and behaviors, and to improve personal hygiene. Consequently, health education is an important strategy to prevent infestation and children need to be made aware of preventive actions they can adopt.

Health education programs (including teacher workshops, video cartoons, posters, pamphlets, and drawing activities) have a beneficial impact on prevention and control of various infectious diseases [15–18]. However, there has been little attention on the effectiveness of health education on the prevalence of pediculosis, and especially whether the rate of head lice infestation can be reduced by changing the behaviors of children. One previous study in Thailand reported a health education program on pediculosis involving a small number of children. The study was statistically inconclusive due to methodological limitations (such as the lack of randomization, small sample size, lack of control group and of advanced data analysis) [19], indicating that better designed studies are required. Any health education program should be aimed at a specific target population and should not interfere with the general classroom teaching.

Here, we report the development and testing of a health education program, in Khon Kaen, Thailand, aimed at prevention and reduction of pediculosis among school girls. The key messages given to the intervention group included: avoid head-to-head contact during play and other activities at home and school; hair should be washed every day; clothing, hats, scarves, coats, combs, sports uniforms, towels and other personal items should not be shared; hair and scalp should be carefully inspected regularly to identify lice or their eggs [20].

## Methods

### Ethics statement

The study was performed after obtaining approval from the Ethical Review Committee of Khon Kaen University, (HE591537). Written informed consent was obtained from each child’s parent or guardian and written child assent was obtained from each child. Permission to study in each school was granted by the school’s director.

### Study area and design

**Fig 1:**
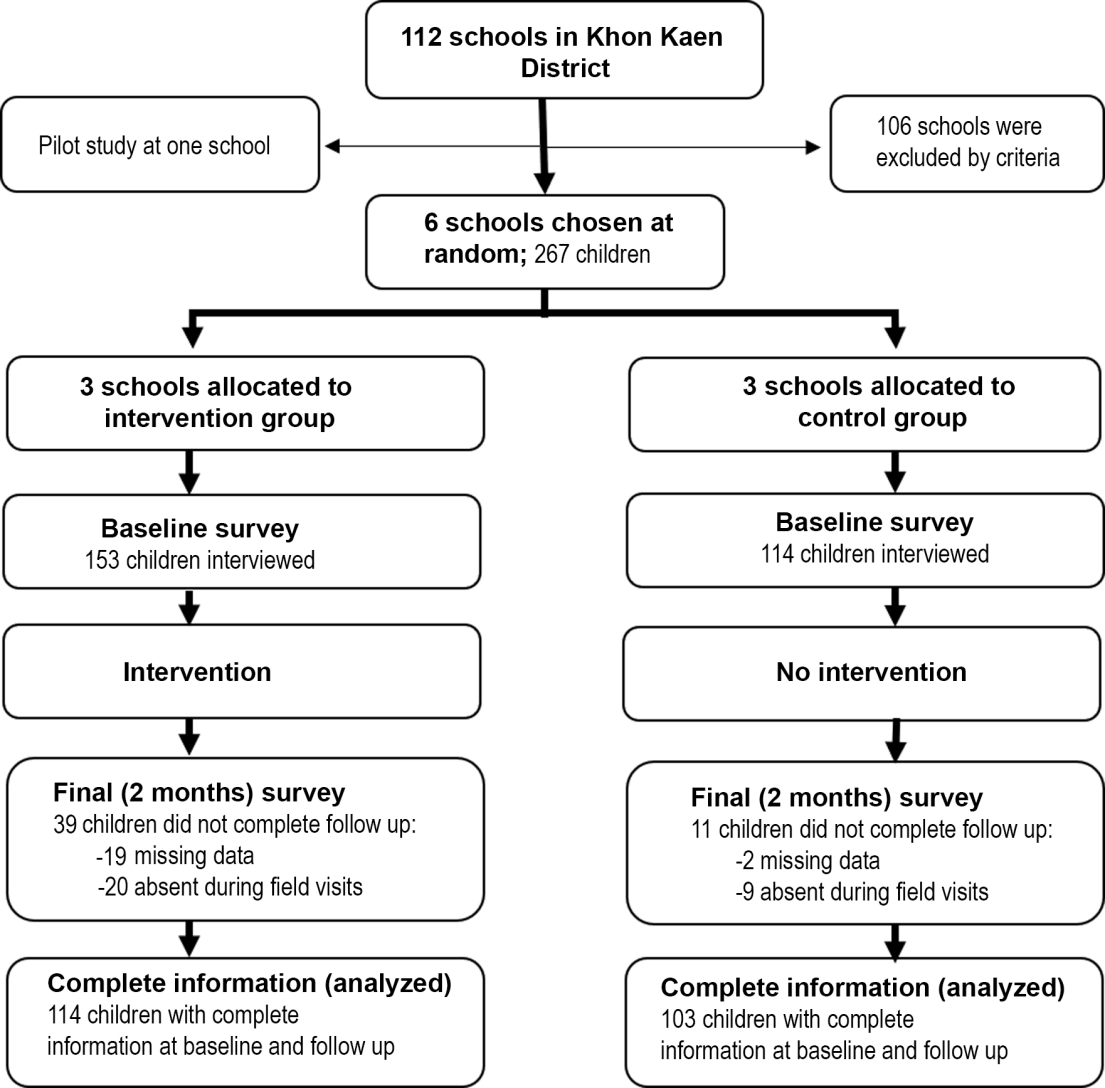
Flow chart for study. Six schools were chosen at random and assigned to intervention or control groups. The health education program was provided to the intervention group. Head lice infestation was investigated at baseline and at two months after intervention.

This study was conducted from January 2017 to August 2017 in primary schools in Muang Khon-Kaen District, Khon Kaen Province, northeastern Thailand. The sample size of primary schools in this area was based on the infestation rate of head lice reported in a study conducted in 1988 [9]. The target population was female schoolchildren aged 4–11 years in kindergarten class levels 1–2 and primary school class levels 1 to 4. Participation of teachers, parents, and their children was entirely voluntary. Parents were required to complete consent forms before their children could be enrolled. Parents were also asked to complete questionnaires detailing relevant demographic, socioeconomic and environmental factors. Knowledge, attitude and practice (KAP) questionnaires and health education materials were prepared, outlining information and key risk factors for head lice transmission and infestation. These materials were checked by various specialists, including parasitologists, medical education technologists and health education experts. Pilot testing of this material was conducted in one school (not included among the intervention and control schools). The health education program was subsequently provided only to the main intervention group (Fig 1). The KAP questionnaire was re-evaluated at two months after intervention. Head lice and nits were removed from children by the researcher and the teacher using a fine-tooth comb.

### Sampling and sample size

Muang District is one of three districts in Khon Kaen Area One (in which there are 112 schools). Schools were selected using multistage cluster sampling and simple randomization. The criteria for selecting schools were: each should be a government school, the school should be large enough to have more than 50 girl children, schools should be only at the primary level. Schools offering secondary-level classes were excluded to avoid possible contamination from older pupils. Based on these criteria, we selected schools located in only in the Muang district.

Three schools in the Savatee Sub-district, two schools in Koksri Sub-district and one school in Donhun Sub-district were selected. Control and intervention groups were drawn from different schools. To prevent contamination between intervention and control groups, each school in one group was more than 10 km from any school in the other. The sample size was determined on primary outcome–the rate of head lice infestation before and after provision of the health education program. The sample needed to be large enough to have 80% power to show a reduction of 20% in the prevalence of head lice infestation, to identify a difference between intervention and control groups, and also to evaluate changes in knowledge, attitude and preventive practice scores after intervention [21]. An overall sample size of 180 individuals (90 in each group) was therefore required. To allow for a drop-out rate of 20%, 216 individuals were required (108 in each group). However, the total population of eligible girl children in the 6 schools was 267 individuals, and it was decided to include all individuals.

### Data collection and measures

At baseline, data collected included prevalence and intensity rates of head lice infestation. Knowledge, attitude and practice (KAP) questionnaires were provided to the children in the intervention group. The prevalence of head lice was determined by the researcher and the teacher using a fine-tooth comb to examine children. Intensity of infestation was scored as high (more than one louse with the first stroke of the comb), medium (one louse with first stroke) or low (one louse found only after 5–6 strokes of the comb) [22].

The questionnaire was administered in two sessions (S1 Table). During the first session, parents were asked about their occupation, demographic characteristics, behaviors, environment and exposure to key risk factors for head lice infestation. The second session involved the children themselves, and focused on their knowledge, attitude and practice (KAP) relating to head lice. The self-administered KAP questionnaire was comprised of five parts. The first part requested information such as age, school level and disease status. The second part contained 12 “yes /no /do not know” questions about head lice. In this part, incorrect and correct answers were scored as zero and one, respectively. The third part contained 10 “agree/ disagree /not sure” questions about attitudes towards head lice. A correct answer was scored as three, the “not sure” answer was scored as two and an incorrect answer was given a score of one. The fourth part collected questions about the health education material provided. The final section enquired about behaviors and practices for preventing pediculosis. Questions in this part were multi-choice with four options (always, sometimes, rarely and never). The content, face and structure validity of the questionnaires were reviewed by specialists in health education and Parasitology.

### Development of the health education material

The health education material was prepared after determination of the behavioral, environmental and key risk factors that may influence head lice infestation (S2 Table). The purposes of the material were to increase knowledge, change attitudes and teach preventive practices. The material included topics such as life cycle of head lice, modes of transmission, signs and symptoms, diagnosis, treatment, prevention and control. Key messages, phrased in simple language suitable for the target population, were added. Also included were teacher-training workshops, preparation of posters, cartoon animations, songs and booklets, development of activities such as drawing activities, distribution of sanitary bags (containing a comb, pamphlet, plastic hair cover and shampoo) and face-to-face discussions.

The health education materials were quality-tested in a pilot study carried out in a school not included in the experimental groups. A total of 15 children in second-level classes of a primary school and 5 teachers were enrolled to test the materials. A short questionnaire was filled out by the audience during the second viewing of the cartoon animation. Their responses were discussed with children and teachers and then the audiences were invited to comment on the health education materials and make suggestions. Their feedback was used to improve the materials.

### Health education intervention

Teachers of the intervention group were given basic knowledge about head lice. A half-day training session for teachers was conducted one week before the first session with schoolchildren. The teachers, in conjunction with the researcher, tested the children’s knowledge and the results were recorded in a book. The story concepts presented in the cartoon animation, song and poster were discussed with the class twice per month by the teachers. The children were checked for head lice every week by their teachers. In addition, children were asked to educate their parents on the importance of prevention and treatment. Children were instructed how to use the comb and shampoo given to them in the sanitary bag, especially when they felt itching of the scalp or had the sensation of something moving in the hair. The health education program was continued until follow-up at two months.

At follow-up, changes in prevalence, incidence (of new cases) and intensity of head lice infestation were calculated. The post-intervention scores on knowledge, attitude and practice were evaluated using KAP questionnaires and compared with the pre-intervention results. After two months, all children with head lice infestations in both the intervention and control groups were treated with 1% malathion.

### Statistical analysis

Descriptive statistics were reported as frequencies and percentages. The characteristics of study sites and demographic, behavioral and socioeconomic data were collected. Chi-square tests were used to compare groups. The efficacy of the health education program was measured by comparing across intervention and control groups pre- and post-intervention using independent-tests and paired-test or analysis of covariance, and each estimator was presented with its mean difference and 95% confidence interval: p values less than 0.05 were considered statistically significant. Changes in prevalence and intensity of infestation were evaluated using chi-square tests. The correlation between knowledge, attitude and practice were compared by multiple linear regression analysis. All statistical analyses were performed using SPSS 16.0.

## Results

A total of 267 children in Amphoe Muang, Khon Kaen Province, enrolled from six randomly selected schools were allocated into intervention (n = 153) and control groups (n = 114) at baseline assessment, as shown in Fig 1. The KAP questionnaire was re-evaluated at two months after intervention. Complete information was obtained and analyzed from a total of 114 children from the intervention group and 103 children from the control group.

At baseline, intervention and control groups (Table 1), were not statistically significantly different in terms of sex, age, status, occupation and educational level of parent (P > 0.05). The overall rate of head lice infestation is shown in Table 2. The prevalence in the intervention and control groups, a difference that was not statistically significant (P > 0.05). Also, the intensities of head lice infestations in intervention and control groups were not statistically significantly different.

**Table 1:** Students’ demographic information in intervention and control groups at baseline.

Demographic variables^a^	Intervention group N (%)	Control group N (%)	P*
**Sex of the parent interviewed**	** **	** **	** **
Male	11 (10.19)	6 (6.38)	0.332
Female	97 (89.81)	88 (93.62)	
**Age of parent**			
<30	14 (13.46)	11 (12.09)	0.502
30–50	73 (73.07)	70 (76.92)	
>50	17 (16.35)	10 (10.99)	
**Status**			
Single	3 (2.78)	0 (0)	0.255
Married	89 (82.41)	81 (86.17)	
Divorce	16 (14.81)	13 (13.83)	
**Parent's level of education**			
Primary school	39 (36.11)	43 (45.74)	0.393
Junior high school	35 (32.41)	22 (23.40)	
Senior high school	31 (28.70)	25 (26.60)	
University education	3 (2.78)	4 (4.26)	
**Parent's occupation**			
Agricultural	53 (49.07)	50 (53.19)	0.491
Worker	12 (11.11)	14 (14.89)	
Teacher	1 (0.93)	2 (2.13)	
Other	42 (38.89)	28 (29.79)	
**History of head lice infestation**			
Previous infestation	78 (72.22)	60 (63.83)	0.082
No previous infestation	29 (26.85)	28 (29.79)	
Don’t know	1 (0.93)	6 (6.38)	

* Statistically significant difference between intervention and control groups based on Chi square test.

^a^ All variables were obtained by interviewing the parents of the children.

**Table 2:** The rate of head lice infestation, incidence and intensity in intervention and control groups at baseline and the two-month follow-up assessment.

Variables	Intervention group	Control group	Percent	P*
	Percentage^a^ (N)	Percentage^a^ (N)	difference^b^	
**Total infestation rate** (presence of eggs, adults and/or nymphs)				
At baseline	59 (90/153)	56 (64/114)	3.00%	0.561
At follow up	44 (51/114)	65 (67/103)	21.00%	0.003
P value**	0.023	0.180		
**Infestation rate of eggs**				
At baseline	15.69 (24/153)	9.65 (11/114)	6.04%	0.149
At follow up	7.89 (9/114)	10.68 (11/103)	2.79%	0.479
P value**	0.056	0.802		
**Intensity of head lice** (adult and nymph)				
At baseline				
High	24.84 (38/153)	21.05 (24/114)	3.35%	0.236
Medium	7.84 (12/153)	15.79 (18/114)		
Low	10.46 (16/153)	9.65 (11/114)		
At follow up				
High	20.18 (23/114)	31.07 (32/103)	17.53%	0.066
Medium	7.89 (9/114)	8.74 (9/103)		
Low	8.77 (10/114)	14.56 (15/103)		
P value**	0.742	0.110		
**Incidence of new infestations**				
At follow up	6.14 (7/114)	12.62 (13/103)		

* Statistically significant difference between intervention and control groups.

** Statistically significant difference before and after intervention.

^a^ All variables are percentages and numbers of infested children.

^b^ Difference in percentage of head lice infestation between intervention and control groups.

At two months post-intervention, the overall prevalence of head lice had decreased to 44% in the intervention group but had increased to 65% in the control group. The intensity of infestation in the intervention group decreased non-significantly between baseline and follow-up assessment (P > 0.05). The percentage of new cases (incidence) was 6.14% in the intervention group and 12.62% in the control group at the two-month follow up.

Differences between the control and intervention groups in knowledge, attitude and preventive practice (KAP) were evaluated pre- and post-intervention (Table 3). The groups did not significantly differ in mean scores at baseline assessment (P > 0.05). In contrast, there was a statistically significant increase in mean scores of KAP in the intervention group at the two-month follow-up (P < 0.05).

**Table 3:** The participants’ mean scores of knowledge, attitude and practice about head lice in intervention and control groups at baseline and at the two months follow-up assessment.

Variables	Intervention group	Control group	Mean difference (95% CI)	P*
	Mean (SD)	Mean (SD)		
**Total mean scores of knowledge**^a^				
At baseline	5.601 (1.55)	5.421 (1.24)	0.18 (-0.169, 0.529)	0.310
At follow up	7.368 (1.61)	5.679 (1.56)	1.69 (1.263, 2.115)	0.001
P value**	0.001	0.205		
**Total mean scores of attitude**^b^				
At baseline	2.355 (0.22)	2.349 (0.26)	0.006 (-0.052, 0.637)	0.844
At follow up	2.637 (0.28)	2.298 (0.25)	0.339 (0.268, 0.411)	0.001
P value**	0.001	0.133		
**Total mean scores of practice**^b^				
At baseline	2.34 (0.74)	2.31 (0.80)	0.039 (-0.146, 0.226)	0.672
At follow up	2.65 (0.52)	2.43 (0.71)	0.218 (0.053. 0.383)	0.010
P value**	0.001	0.538		

* Statistically significant difference between intervention and control groups.

** Statistically significant difference before and after intervention.

^a^ Score out of 12.

^b^ Score out of 3.

The mean scores of changing attitude pre- and post-intervention are presented in Table 4, based on independence-tests. Ten questions were compared. For most of these, there was no significant difference between intervention and control groups at baseline (P > 0.05). At the two-month follow-up, the scores for most questions had increased significantly in the intervention group (P < 0.05). However, the scores for “sharing personal items can cause infestation with head lice” did not significantly differ between intervention and control groups at the end of the study (P = 0.054).

**Table 4:** Attitudes about head lice in intervention and control groups at baseline and at the two-month follow-up assessment.

Variables	At baseline assessment				At follow up assessment			
	Intervention N = 153 (%) ^a^	Control N = 114 (%) ^a^	Mean dif^b^	P*	Intervention N = 114 (%) ^a^	Control N = 103 (%) ^a^	Mean dif^b^	P*
**Are head lice nasty insect?**								
Agree	143 (93.46)	104 (91.23)	0.423	0.372	113 (99.10)	96 (93.20)	0.079	0.021
Disagree	6 (3.92)	8 (7.02)			0	2 (1.94)		
Not sure	4 (2.61)	2 (1.75)			1 (0.90)	5 (4.85)		
**Head lice can be cured.**								
Agree	117 (76.47)	85 (74.56)	0.046	0.626	100 (87.70)	73 (70.87)	0.331	0.001
Disagree	24 (15.69)	21 (18.42)			8 (7.00)	24 (23.30)		
Not sure	12 (7.84)	8 (7.02)			6 (5.26)	6 (5.83)		
**Can prevent head lice infestation by hair washing?**								
Agree	137 (89.54)	99 (86.84)	0.051	0.423	111 (97.37)	87 (84.47)	0.199	0.002
Disagree	7 (4.58)	8 (7.02)			2 (1.75)	9 (8.74)		
Not sure	9 (5.88)	7 (6.14)			1 (0.88)	7 (6.80)		
**Head lice can be acquired by sharing personal items.**								
Agree	133 (86.93)	97 (85.09)	0.043	0.557	103 (90.35)	81 (78.64)	0.164	0.054
Disagree	11 (7.19)	11 (9.65)			9 (7.89)	13 (12.62)		
Not sure	9 (5.88)	6 (5.26)			2 (1.75)	9 (8.74)		
**Pediculicidal compounds can kill all stages of head lice.**								
Agree	117 (76.47)	88 (77.19)	0.022	0.809	39 (34.20)	67 (65.05)	0.640	0.001
Disagree	21 (13.73)	19 (16.67)			61 (53.52)	19 (18.45)		
Not sure	15 (9.80)	7 (6.14)			14 (12.3)	17 (16.50)		
**Head lice should not be treated.**								
Agree	20 (13.07)	15 (13.16)	0.037	0.662	8 (7.00)	35 (34.00)	0.558	0.001
Disagree	56 (36.60)	46 (40.35)			96 (84.20)	57 (55.30)		
Not sure	77 (50.33)	53 (46.49)			10 (8.77)	11 (10.70)		
**Prevention of head lice is easy.**								
Agree	102 (66.67)	75 (65.79)	0.067	0.490	87 (76.32)	60 (58.25)	0.239	0.012
Disagree	22 (14.38)	23 (20.18)			11 (9.65)	16 (15.53)		
Not sure	29 (18.95)	16 (14.04)			16 (14.04)	27 (26.21)		
**The itching caused by head lice can make you lose concentration while studying.**								
Agree	84 (54.90)	69 (60.53)	0.201	0.043	94 (82.46)	68 (66.02)	0.256	0.014
Disagree	41 (26.80)	14 (12.28)			15 (13.16)	23 (22.33)		
Not sure	28 (18.30)	31 (27.19)			5 (4.39)	12 (11.65)		
**Head lice can make you sick.**								
Agree	106 (69.28)	83 (72.81)	0.027	0.776	76 (66.66)	49 (47.57)	0.364	0.001
Disagree	20 (13.07)	22 (19.30)			19 (16.67)	35 (33.98)		
Not	27 (17.65)	9 (7.89)			19 (16.67)	19 (18.45)		
**If you have head lice your friends will not play with you.**								
Agree	111 (72.55)	86 (75.44)	0.030	0.742	55 (48.20)	76 (73.79)	0.567	0.001
Disagree	23 (15.03)	17 (14.91)			51 (44.7)	14 (13.59)		
Not sure	19 (12.42)	11 (9.65)			8 (7.02)	13 (12.62)		

* Statistically significant difference between intervention and control groups

^a^ All variables are number of children and percentages

^b^ Difference in mean attitude scores between intervention and control groups.

Preventive practices, for instance hair washing, clothes washing, asking parents to check the head when it itches, and sharing personal items, were evaluated (Table 5). The mean scores did not significantly differ between intervention and control groups at baseline assessment (P > 0.05). In contrast, the mean scores for four preventive practices had significantly improved in the intervention group at the two-month follow-up. Only the scores relating to preventive practice in terms of clothes washing did not significantly differ between the groups at the two-month follow-up (P = 0.653).

**Table 5:** The frequency of preventive practices in intervention and control groups at baseline and the two-month follow-up assessment.

Variables	At baseline assessment				At follow up assessment			
	Intervention^a^ N = 153 (%)	Control^a^ N = 114 (%)	Mean dif^b^	P*	Intervention^a^ N = 114 (%)	Control^a^ N = 103 (%)	Mean dif^b^	P*
**Washing hair (per week)**	** **	** **	** **	** **	** **	** **	** **	** **
Always (Daily)	78 (51.0)	57 (50.0)	0.083	0.315	70 (61.4)	43 (41.7)	0.212	0.011
Sometimes (3–4 times)	66 (43.1)	44 (38.6)			38 (33.3)	54 (52.4)		
Rarely (1–2 times)	9 (5.9)	11 (9.6)			6 (5.3)	5 (4.9)		
Never	0	2 (1.8)			0	1 (1.0)		
**Washing clothes (per month)**								
Always Every week)	45 (29.4)	32 (28.1)	0.026	0.842	39 (34.2)	43(41.7)	0.071	0.653
Sometimes (3 times)	46 (30.1)	37 (32.5)			43 (37.7)	24 (23.3)		
Rarely (1–2 times)	40 (26.1)	26 (22.8)			11 (9.6)	9 (8.7)		
Never	22 (14.4)	19 (16.7)			21 (18.4)	27 (26.2)		
**Asking parent to check head**								
Always	101 (66.0)	76 (66.7)	0.013	0.620	69 (60.5)	58 (56.3)	0.269	0.037
Sometimes (3–4 times)	24 (15.7)	14 (12.3)			36 (31.6)	23 (22.3)		
Rarely (1–2 times)	12 (7.8)	13 (11.4)			4 (3.5)	8 (7.8)		
Never	16 (10.5)	11 (9.6)			5 (4.4)	14 (13.6)		
**Sharing personal items**								
Always	0	2 (1.8)	0.039	0.605	0	0	0.174	0.012
Sometimes (3–4 times)	11 (7.2)	9 (7.9)			0	11 (10.7)		
Rarely (1–2 times)	11 (7.2)	5 (4.4)			10 (8.8)	5 (4.9)		
Never	131 (85.6)	98 (86.0)			104 (91.2)	87 (84.5)		

* Statistically significant difference between intervention and control groups.

^a^ All variables are numbers of children and percentages.

^b^ Difference in means of practice scores between intervention and control groups

The Pearson’s correlation coefficient between knowledge, attitude, practice and classroom teaching mean scores in the intervention group at the two-month follow-up assessment is shown in S3 Table. Preventive practices were significantly correlated with attitude and knowledge in the intervention group. Teaching at school was also positively correlated with preventive practices in the intervention group (P < 0.01). The association of factors leading to a change in personal hygiene behavior was evaluated. Three factors (knowledge, attitude and classroom teaching) could predict behavior change by 26.20% (R = 0.262, P <0.001) in the intervention group (S4 Table).

## Discussion

Head lice infestation is commonly found among schoolchildren, especially girls, and its prevalence is still high worldwide [1]. In Thailand, although a pediculicide treatment strategy has long been used for schoolchildren, pediculosis persists. One explanation is that some pediculicide treatments affect only adult insects, and the remaining nits contribute to re-infestation [14]. Recently, Moshki et al., successfully established a health education program in Iran as an alternative approach to combating pediculosis. Improved KAP was observed among members of the intervention group. However, no assessment of actual prevalence of pediculosis was made before or after the intervention [8]. Furthermore, provision of the health education program disrupted the schedule of teaching activities and the program could not be offered as a main subject for preschool children [23]. Thus, in this study, we have established a new health education program that can specifically target children at various stages of their education. Health education materials were developed to create an appropriate program for several age ranges. Our program was also adapted for use during normal classroom teaching.

The study enrolled schoolchildren at Amphoe Muang Khon Kaen, Thailand. All schools were in a suburban area. Demographic, socioeconomic and behavioral characteristics as well as the history of head lice infestation did not differ significantly between intervention and control groups at baseline. A high overall prevalence of pediculosis (58.60%, n = 267) was found at the baseline survey: 59% of participants in the intervention group and 56% in the control group were infested. This suggests an increase in prevalence in Khon Kaen Province since a previous study in 1988 (44%) [9]. Reported prevalences of head lice among school girls children in rural areas across Thailand for the period 1988–2016 range from 15.10% to 86.12% [9, 12, 13, 24].

In the current study, the impact of a health education program to reduce head lice infestation was evaluated. Although the intensity of head lice (nymphs and adults) in the intervention group decreased greatly after intervention, this was not statistically significant. The incidence of new cases of pediculosis was 6.14% in the intervention group and 12.62% in the control group at the two-month follow-up. Most new cases occurred in the kindergarten class levels, perhaps because children at these levels sleep in the afternoons and thus have contact with bedding. However, the health education program was shown to be effective in reducing overall head lice infestation from 59% to 44% (by 15%) in the intervention group, a significant difference compared to the control group, when egg-only infestations are included. Indeed, the prevalence of pediculosis in the control group increased from 56% to 65% at the two-month follow-up, implying that without any prevention and control program, the rate of infestation will continually increase. The positive results of our health education trial were similar to those reported in previous studies [23]. For example, the prevalence of pediculosis among schoolchildren in Iran decreased from 69.30 to 26.70% after a one-month intervention. Similarly, the prevalence of head lice infestation in an intervention group in Egypt was reduced from 44.20% to 7.20% after intervention and treatment [25]. The reduction of head lice infestation in our study was not as great as that in Egypt. This might be due to the survival of nits and adults in some individuals. Therefore, we suggest that a combination of health education together with pediculicide treatment should be used to achieve effective and sustainable pediculosis control.

Our health education program significantly increased mean knowledge scores. In relation to attitudes, only the choice *“*head lice can be acquired by sharing personal items” was not statistically different between the two groups after intervention. Frequency of preventive practices (washing hair and telling parents when itching) in the intervention group was significantly increased post-intervention. On the other hand, there was no significant difference between the groups after intervention in the practice of washing clothes. This is probably because children at the kindergarten levels did not wash their own clothes. Our health education program produced an overall increase in the total mean scores of KAP in the intervention group but not in the control group.

The beneficial effect of increased knowledge and improved attitudes towards personal hygiene has been shown by many previous studies [19, 23, 26–28]. We also found a correlation between knowledge, attitude and preventive practices in the intervention group. Furthermore, relevant classroom teaching was also correlated with preventive practice. We designed a schedule for checking personal hygiene of the children every week (S5 Table). Children with good personal hygiene were rewarded with affective scores or a gift. This approach is based on the operant-conditioning theories of B.F. Skinner [29], rewarding children who have positive practices. Thus, the school teacher is an important person to transfer information about prevention and control of the disease to the children [30–32]. Indeed, the knowledge, attitude and teaching of the teacher predicted 26.20% of observed behavior change at two months in the intervention group. In another study, the change after one month was only 7.40% [8].

Factors such as age of children, educational levels of parents and children, parental occupation, family income, number of siblings, number sleeping in the same room, demographics, behavioral habits and socioeconomic status can be correlated with the rate of head lice infestation [10, 12, 24, 33–35], but may vary by endemic area. Thus, these factors should be also considered when establishing strategies for prevention and control of pediculosis.

This study had some limitations. We only included children from a few schools in the study area. Contact between children from different schools could lead to transfer of head lice. Sustainable eradication of pediculosis should use combinations of health education programs and a safe drug that can be administered at schools and in the community. Pediculicidal products are very expensive and have side effects. In developing countries, including Thailand, both of these issues worry parents [36]. Therefore, the government should support a search for new, safe drugs, perhaps including traditional drugs [37–39]. A strength of this study is that the health education program could be used to increase knowledge, change attitudes and improve preventive practices among schoolchildren, thus reducing rates of infestation without necessarily involving the use of pediculicides. The health education program did not interrupt teaching activity. Such educational materials should be available in the classroom and in the community. Long-term investment in this program should lead to sustainable prevention of head lice infestation.

## Conclusion

Head lice infestation is still highly prevalent among schoolchildren in Amphoe Muang, Khon Kaen Province. The health education program described here was successful in increasing knowledge, changing attitudes towards preventive practice and in reducing the rate of infestation over a two-month period.

## Supporting information

S1 Table Questionnaires. (PDF)

S2 Table Poster. (TIF)

S3 Table The correlation of knowledge, attitude, practice and teacher in the intervention group at two months follow-up assessment. (PDF)

S4 Table The prediction of association of factors to change personal hygiene in the intervention group at two months follow-up assessment. (PDF)

S5 Table Appendix. (PDF)
