# Supplementary material for: Effect of a health education program on reduction of pediculosis in school girls at Amphoe Muang, Khon Kaen Province, Thailand
Source: PLoS One. 2018 Jun 11;13(6):e0198599. doi: 10.1371/journal.pone.0198599 (PMC5995376; doi:10.1371/journal.pone.0198599)
Supplement: S1 Table — (PDF) [file pone.0198599.s001.pdf]

ID School…………

The questionnaire for the parents

Title: Development of health education program on pediculosis in girl school children at Amphoe Muang, Khon Kaen Province, Thailand

**<u>This part for the researcher</u>**
SC……………………………CL………………..GL………………Code No………………….

Part 1. General information of parent

The next few questions will ask you about your family, your activities and habits. Please check ✓inside ❒ the most suitable answer.

1. Sex

❒ 1. Male ❒ 2. Female

2. Age………………..………year

3. Status

❒ 1. Single ❒ 2. Married ❒ 3. Divorce

4. Parent's level of education

❒ 1. Primary school ❒ 2. Junior high school

❒ 3. Senior high school ❒ 4. University education

❒ 5. Other …………………………

5. Parent's occupation

❒ 1. Agricultural

❒ 2. Worker

❒ 3. Teacher

❒ 4 Other …………………………………

6. History of head lice infestation

❒ 1. Yes ❒ 2. No ❒ 3. Do not know

Part 2. Risk factor of head lice infestation

Please check ✓inside ❒ the most suitable answer.

1. The frequency of washing clothes?

❒ 1. At least once a week ❒ 2 Twice a month ❒ 3. Rarely ❒ 4. Never

2. Do you sleep in the same room as your daughter?

❒ 1. Yes ❒ 2. No

3. Do you share personal items with your daughter?

❒ 1. Yes ❒ 2. No

4. The frequency of checking the head of your daughter when they itching?

❒ 1. Weekly ❒ 2. Sometimes ❒ 3. Rarely ❒ 4. Never

Thank you

ID School…………

The questionnaire for the primary girl school children

(At baseline)

Title: Development of health education program on pediculosis in girl school children at Amphoe Muang, Khon Kaen Province, Thailand

**This part for the researcher**
SC……………………………CL………………..GL……………….Code No………………….

Part 1. General information of children

Please check ✓inside ❒ the most suitable answer.

Do you have any medications?

❒ 1. No ❒ 2. Yes ……………………………………

Part 2. Knowledge about head lice

Please check ✓inside the most suitable answer.

**Statement**	**Yes**	**No**	**I do not know**
1. Head lice can jump.			
2. Head lice can survive several days on clothes and furniture.			
3. Head lice crawl from head to head in close contact.			
4. People getting head lice always start to itch immediately.			
5. Head lice will survive an ordinary shampooing.			
6. Some available pediculicides kill all lice eggs.			
7. Only persons having head lice should be treated with pediculicides.			
8. The home must be thoroughly cleaned if head lice are found.			
9. Head lice can spread from pets or farm animals.			
10. Head lice spread easily from pillows, furniture, animal fur and clothes.			
11. Treatment with pediculicides must be done twice.			
12. Persons having head lice and who are not treated may infest others repeatedly.			

ID School…………

Part 3. Attitude about head lice

Please check ✓inside the most suitable answer.

Statement	Agree	Disagree	Not sure
1. Are head lice nasty insect?			
2. Head lice infestations can be cured?			
3. Head lice infestations can be prevented by hair washing?			
4. Head lice can be transmitted to others by sharing personal items?			
5. Pediculicidal treatment can kill all stage of head lice?			
6. Head lice infestations should not be treated?			
7. Prevention head lice infestation is easy?			
8. The itching caused by head lice can make you lose concentration while studying?			
9. Head lice can make you sick?			
10. If you have head lice your friend will not play with you?			

Part 4. Evaluation of health education program

Please check ✓inside ❒ the most suitable answer.

1. What are the sources of information about head lice that you best understand?

   ❒ 1. Cartoon ❒ 2. Poster ❒ 3. Teacher ❒ 4. Other………❒ 5. Don't remember

2. The teacher talks to you about head lice

   ❒ 1. Always (Every week) ❒ 2. Sometimes (twice a month) ❒ 3. Never

3. How much do you think you understand about prevention of head lice?

   ❒ 1. A lot ❒ 2. Some ❒ 3. Little

Part 5. The frequency of preventive practices

Please check ✓inside ❒ the most suitable answer.

1. The frequency of washing hair per week?

   ❒ 1. Always (Daily) ❒ 2.Sometimes (3-4 times per week)

   ❒ 3. Rarely (1-2 per week) ❒ 4. Never

2. The frequency of washing clothes help your parents?

   ❒ 1. Always (Every week) ❒ 2.Sometimes (3 times per month)

   ❒ 3. Rarely (1-2 times per month) ❒ 4. Never

3. The frequency of asking parent to check the head when it itches?

   ❒ 1. Always ❒ 2.Sometimes (3-4) ❒ 3. Rarely (1-2) ❒ 4. Never

4. The frequency of sharing personal items together?

   ❒ 1. Always ❒ 2.Sometimes (3-4) ❒ 3. Rarely (1-2) ❒ 4. Never

Thank you

ID School…………

The questionnaire for the primary girl school children

(At follow up)

Title: Development of health education program on pediculosis in girl school children at Amphoe Muang, Khon Kaen Province, Thailand

This part for the researcher

SC……………………………CL………………..GL………………Code No………………….

Part 1. General information of children

Please check ✓inside ❐ the most suitable answer.

Do you have any medications?

❐ 1. No ❐ 2. Yes as……………………………………

Part 2. Knowledge about head lice

Please check ✓inside the most suitable answer.

Statement	Yes	No	I do not know
1. Head lice can jump.			
2. Head lice can survive several days on clothes and furniture.			
3. Head lice crawl from head to head in close contact.			
4. People getting head lice always start to itch immediately.			
5. Head lice will survive an ordinary shampooing.			
6. Some available pediculicides kill all lice eggs.			
7. Only persons having head lice should be treated with pediculicides.			
8. The home must be thoroughly cleaned if head lice are found.			
9. Head lice can spread from pets or farm animals.			
10. Head lice spread easily from pillows, furniture, plush animals and clothes.			
11. Treatment with pediculicides must be done twice.			
12. Persons having head lice and who are not treated may infest others repeatedly.			

ID School…………

Part 3. Attitude about head lice

Please check ✓inside the most suitable answer.

Statement	Agree	Disagree	Not sure
1. Are head lice nasty insect?			
2. Head lice infestations can be cured?			
3. Head lice infestations can be prevented by hair washing?			
4. Head lice can be transmitted to others by sharing personal items?			
5. Pediculicidal treatment can kill all stage of head lice?			
6. Head lice infestations should not be treated?			
7. Prevention head lice infestation is easy?			
8. The itching caused by head lice can make you lose concentration while studying?			
9. Head lice can make you sick?			
10. 10. If you have head lice your friend will not play with you?			

Part 4. Evaluation of health education program

Please check ✓inside ❐ the most suitable answer.

1. What are the sources of information about head lice that you best understand?

   ❐ 1. Cartoon ❐ 2. Poster ❐ 3. Teacher ❐ 4. Other………❐ 5. Don't remember

2. The teacher talks to you about head lice

   ❐ 1. Always (Every week) ❐ 2. Sometimes (twice a month) ❐ 3. Never

3. How much do you think you understand about prevention of head lice?

   ❐ 1. A lot ❐ 2. Some ❐ 3. Little

Part 5. The frequency of preventive practices

Please check ✓inside ❐ the most suitable answer.

1. The frequency of washing hair per week?

   ❐ 1. Always (Daily) ❐ 2.Sometimes (3-4 times per week)

   ❐ 3. Rarely (1-2 per week) ❐ 4. Never

2. The frequency of washing clothes help your parents?

   ❐ 1. Always (Every week) ❐ 2.Sometimes (3 times per month)

   ❐ 3. Rarely (1-2 times per month) ❐ 4. Never

3. The frequency of asking parent to check the head when it itches?

   ❐ 1. Always ❐ 2.Sometimes (3-4) ❐ 3. Rarely (1-2) ❐ 4. Never

4. The frequency of sharing personal items together?

   ❐ 1. Always ❐ 2.Sometimes (3-4) ❐ 3. Rarely (1-2) ❐ 4. Never

Thank you

ID School…………

The questionnaire for the kindergarten girl school children
(At baseline)

Title: Development of health education program on pediculosis in girl school children at Amphoe Muang, Khon Kaen Province, Thailand

This part for the researcher

SC……………………………CL………………..GL……………….Code No………………….

Part 1. General information of children

Please check ✓inside ❒ the most suitable answer.

Do you have any medications?

❒ 1. No ❒ 2. Yes as……………………………………

Part 2. Knowledge about head lice

Please check ✓inside the most suitable answer.

Statement	Yes	No	I do not know
1. Head lice can jump.			
2. Head lice can survive several days on clothes and furniture.			
3. Head lice crawl from head to head in close contact.			
4. People getting head lice always start to itch immediately.			
5. Head lice will survive an ordinary shampooing.			
6. Some available pediculicides kill all lice eggs.			
7. Only persons having head lice should be treated with pediculicides.			
8. The home must be thoroughly cleaned if head lice are found.			
9. Head lice can spread from pets or farm animals.			
10. Head lice spread easily from pillows, furniture, plush animals and clothes.			
11. Treatment with pediculicides must be done twice.			
12. Persons having head lice and who are not treated may infest others repeatedly.			

ID School…………

Part 3. Attitude about head lice

Please check ✓inside the most suitable answer.

Statement	Agree	Disagree	Not sure
1. Are head lice nasty insect?			
2. Head lice infestations can be cured?			
3. Head lice infestations can be prevented by hair washing?			
4. Head lice can be transmitted to others by sharing personal items?			
5. Pediculicidal treatment can kill all stage of head lice?			
6. Head lice infestations should not be treated?			
7. Prevention head lice infestation is easy?			
8. The itching caused by head lice can make you lose concentration while studying?			
9. Head lice can make you sick?			
10. 10. If you have head lice your friend will not play with you?			

Part 4. Evaluation of health education program

Please check ✓inside ❒ the most suitable answer.

1. What are the sources of information about head lice that you best understand?

   ❒ 1. Cartoon ❒ 2. Poster ❒ 3. Teacher ❒ 4. Other………❒ 5. Don't remember

2. The teacher talks to you about head lice

   ❒ 1. Always (Every week) ❒ 2. Sometimes (twice a month) ❒ 3. Never

3. How much do you think you understand about prevention of head lice?

   ❒ 1. A lot ❒ 2. Some ❒ 3. Little

Part 5. The frequency of preventive practices

Please check ✓inside ❒ the most suitable answer.

1. The frequency of washing hair per week?

   ❒ 1. Always (Daily) ❒ 2.Sometimes (3-4 times per week)

   ❒ 3. Rarely (1-2 per week) ❒ 4. Never

2. The frequency of washing clothes help your parents?

   ❒ 1. Always (Every week) ❒ 2.Sometimes (3 times per month)

   ❒ 3. Rarely (1-2 times per month) ❒ 4. Never

3. The frequency of asking parent to check the head when it itches?

   ❒ 1. Always ❒ 2.Sometimes (3-4) ❒ 3. Rarely (1-2) ❒ 4. Never

4. The frequency of sharing personal items together?

   ❒ 1. Always ❒ 2.Sometimes (3-4) ❒ 3. Rarely (1-2) ❒ 4. Never

Thank you

ID School…………

The questionnaire for the kindergarten girl school children

(At follow up)

Title: Development of health education program on pediculosis in girl school children at Amphoe Muang, Khon Kaen Province, Thailand

This part for the researcher

SC……………………………CL………………..GL……………….Code No………………….

Part 1. General information of children

Please check ✓inside ❒ the most suitable answer.

Do you have any medications?

❒ 1. No ❒ 2. Yes as……………………………………

Part 2. Knowledge about head lice

Please check ✓inside the most suitable answer.

Statement	Yes	No	I do not know
1. Head lice can jump.			
2. Head lice can survive several days on clothes and furniture.			
3. Head lice crawl from head to head in close contact.			
4. People getting head lice always start to itch immediately.			
5. Head lice will survive an ordinary shampooing.			
6. Some available pediculicides kill all lice eggs.			
7. Only persons having head lice should be treated with pediculicides.			
8. The home must be thoroughly cleaned if head lice are found.			
9. Head lice can spread from pets or farm animals.			
10. Head lice spread easily from pillows, furniture, plush animals and clothes.			
11. Treatment with pediculicides must be done twice.			
12. Persons having head lice and who are not treated may infest others repeatedly.			

ID School…………

Part 3. Attitude about head lice

Please check ✓inside the most suitable answer.

Statement	Agree	Disagree	Not sure
1. Are head lice nasty insect?			
2. Head lice infestations can be cured?			
3. Head lice infestations can be prevented by hair washing?			
4. Head lice can be transmitted to others by sharing personal items?			
5. Pediculicidal treatment can kill all stage of head lice?			
6. Head lice infestations should not be treated?			
7. Prevention head lice infestation is easy?			
8. The itching caused by head lice can make you lose concentration while studying?			
9. Head lice can make you sick?			
10. 10. If you have head lice your friend will not play with you?			

Part 4. Evaluation of health education program

Please check ✓inside ❒ the most suitable answer.

1. What are the sources of information about head lice that you best understand?

   ❒ 1. Cartoon ❒ 2. Poster ❒ 3. Teacher ❒ 4. Other………❒ 5. Don't remember

2. The teacher talks to you about head lice

   ❒ 1. Always (Every week) ❒ 2. Sometimes (twice a month) ❒ 3. Never

3. How much do you think you understand about prevention of head lice?

   ❒ 1. A lot ❒ 2. Some ❒ 3. Little

Part 5. The frequency of preventive practices

Please check ✓inside ❒ the most suitable answer.

1. The frequency of washing hair per week?

   ❒ 1. Always (Daily) ❒ 2.Sometimes (3-4 times per week)

   ❒ 3. Rarely (1-2 per week) ❒ 4. Never

2. The frequency of washing clothes help your parents?

   ❒ 1. Always (Every week) ❒ 2.Sometimes (3 times per month)

   ❒ 3. Rarely (1-2 times per month) ❒ 4. Never

3. The frequency of asking parent to check the head when it itches?

   ❒ 1. Always ❒ 2.Sometimes (3-4) ❒ 3. Rarely (1-2) ❒ 4. Never

4. The frequency of sharing personal items together?

   ❒ 1. Always ❒ 2.Sometimes (3-4) ❒ 3. Rarely (1-2) ❒ 4. Never

Thank you
