# Supplementary material for: Effect of a health education program on reduction of pediculosis in school girls at Amphoe Muang, Khon Kaen Province, Thailand
Source: PLoS One. 2018 Jun 11;13(6):e0198599. doi: 10.1371/journal.pone.0198599 (PMC5995376; doi:10.1371/journal.pone.0198599)
Supplement: S3 Table — (PDF) [file pone.0198599.s003.pdf]

S3 Table. The correlation of knowledge, attitude, practice and teacher in the intervention group at two months follow-up assessment.

**Variables**	**Correlation coefficients (r) ****	***P****
Knowledge - Practice	0.215	0.011
Attitude - Practice	0.384	0.001
Teacher - Practice	0.388	0.001
Classroom teaching - Attitude	0.560	0.001

**Correlation significant at 0.01 level (2 tailed).*

***Pearson's correlation coefficients*
