# Supplementary material for: Effect of a health education program on reduction of pediculosis in school girls at Amphoe Muang, Khon Kaen Province, Thailand
Source: PLoS One. 2018 Jun 11;13(6):e0198599. doi: 10.1371/journal.pone.0198599 (PMC5995376; doi:10.1371/journal.pone.0198599)
Supplement: S4 Table — (PDF) [file pone.0198599.s004.pdf]

S4 Table. The prediction of association of factors to change personal hygiene in the intervention group at two months follow-up assessment.

**Predictor variables**	**Unstandardized B**	**Standardized B**	**Std. Error**	**t**	***P***
(Constant)	-0.811		0.574	-1.413	0.160
Attitude	0.655	0.296	0.222	2.953	0.004
Knowledge	0.105	0.270	0.032	3.252	0.002
Classroom teaching	0.29	0.232	0.124	2.344	0.021

B: Regression coefficient; R2 =0.262, P= 0.001
