# Supplementary material for: Effect of a health education program on reduction of pediculosis in school girls at Amphoe Muang, Khon Kaen Province, Thailand
Source: PLoS One. 2018 Jun 11;13(6):e0198599. doi: 10.1371/journal.pone.0198599 (PMC5995376; doi:10.1371/journal.pone.0198599)
Supplement: S5 Table — (PDF) [file pone.0198599.s005.pdf]

S5 Table.
Appendix 1: Head lice investigation Form

Assign personal ID to each student.
School name……………………………………………class……………Teacher name……………………………………........

IDs	Name of children	Head lice investigation of the children							
		June 2017				July 2017			
		1 week	2 weeks	3 weeks	4 weeks	1 week	2 weeks	3 weeks	4 weeks
**Example:**	**Mana Jaidee**	Egg +adults	Found egg	Found egg	Not found	Not found	Not found	Not found	Not found

Appendix 2: Intervention Details (May to July 2017)

School name……………………………………………….class………….………Teacher name……….………………………..…………

Instruction essay competition: Should provide this program 2 times per month

No	Activities (Intervention school)	Duration time
1	- Check IDs<br>- Investigation of head lice infestation by the teacher.	5 min
2	- Reinforce member to children about head lice information.	1 min
3	- Cartoon shown first time	5 min
4	- Music song dancing<br>- Questions for school children are distributed and explained by teacher<br>- Best drawings receive price	5 min
5	- Cartoon shown second time<br>- Explain posters	3 min
6	- Teacher discusses students’ answers and their questions.<br>- Tell parent to kill head lice by sanitary bag<br>- Best drawings receive price and are hung up in school	1 min
	**Total**	20 min

Appendix 3: Intervention Details (Cont.)

Instruction: Teacher please check ✓ if you have completed the procedure

Activities	June 2017		July 2017	
	1-2 weeks	3-4 weeks	1-2 weeks	3-4 weeks
1. Investigation of head lice infestation				
2. Cartoon shown				
3. Poster shown				
4. Discussion students' questions and answers to receive price.				
